# An increase of larval rearing temperature does not affect the susceptibility of *Phlebotomus sergenti* to *Leishmania tropica* but effectively eliminates the gregarine *Psychodiella sergenti*

**DOI:** 10.1186/s13071-016-1841-6

**Published:** 2016-10-18

**Authors:** Magdalena Jancarova, Jana Hlavacova, Jan Votypka, Petr Volf

**Affiliations:** Department of Parasitology, Faculty of Science, Charles University, Prague, Czech Republic

**Keywords:** *Phlebotomus sergenti*, *Psychodiella sergenti*, *Leishmania tropica*, Vector competence, Effect of temperature, Gregarines

## Abstract

**Background:**

In mosquitoes, it has previously been shown that rearing conditions of immature stages have an effect on the vector competence of adults. Here, we studied the impact of different larval rearing temperatures (27 °C *versus* 32 °C) on the sand fly *Phlebotomus sergenti* Parrot, 1917 and its susceptibility to two parasites: *Leishmania tropica* Wright, 1903, a dixenous trypanosomatid transmissible from sand flies to humans, and *Psychodiella sergenti* Lantova, Volf & Votypka, 2010, a monoxenous sand fly gregarine.

**Results:**

Increased rearing temperature (32 °C) affected the larval developmental times and size of *P. sergenti* adults but had no effect on the susceptibility of *P. sergenti* to *L. tropica*. No differences were found in *Leishmania* infection rates or in the intensities of *Leishmania* infection. Interestingly, increased larval rearing temperature significantly suppressed the development of gregarines. All 117 control sand flies tested were infected with *Ps. sergenti*, and the mean number of gamonts per individual was 29.5. In contrast, only three of 120 sand flies maintained at 32 °C were infected and the mean number of gamonts per individual was just 0.04.

**Conclusions:**

We demonstrated that the increased rearing temperature of *P. sergenti* larvae had no impact on the development of *L. tropica* in adult sand flies but had a profound effect on the gregarine *Ps. sergenti*. We suggest that increasing the larval rearing temperature by 5 °C is a simple and effective way to clean sand fly colonies infected by gregarines.

## Background

Phlebotomine sand flies (Diptera: Psychodidae) are blood-sucking insects traditionally divided into three main genera: *Phlebotomus*, *Lutzomyia* and *Sergentomyia*. They occur in a wide variety of habitats from deserts to rainforests. Both sexes feed on natural sugar sources, such as the sap of plants or honeydew, and females also feed on the blood of a wide range of hosts including humans (reviewed by [1, 2]). Eggs are laid on a substrate rich in organic content. Larval development includes four instars, and usually lasts three to four weeks. The pupa stage usually takes from seven to ten days (reviewed by [3]).

Phlebotomine sand flies are known vectors of bacteria (e.g. *Bartonella bacilliformis*), viruses (mainly genus *Phlebovirus*) and *Leishmania* spp., digenetic parasites causing a variety of symptoms ranging from mild cutaneous lesions to mucocutaneous form to fatal visceral disease (reviewed by [2]). *Leishmania* life-cycle involves intracellular amastigotes in the vertebrate host and extracellular promastigotes in the vector.

Sand flies also harbour their own monoxenous parasites, such as gregarines of the genus *Psychodiella* Votypka, Lantova & Volf 2009 (Apicomplexa: Eugregarinorida), which is found in *Phlebotomus sergenti* (reviewed by [4]). The typical life-cycle of these gregarines starts by the infection of the first-instar larvae by oocysts, which contain sporozoites. In the midgut, the sporozoites escape, attach to the epithelial cells and develop into trophozoites. Later, mature-stage gamonts located either in the gut lumen of the larvae or the haemocoel of adults, undergo sexual development: two complementary gamonts associate in syzygy and form a gametocyst with oocysts inside. In sand fly females, gametocysts stick to the accessory glands and the oocysts are inoculated into the gland lumen to contaminate the surface of eggs (reviewed by [4]). This typical life-cycle is modified in *Psychodiella sergenti*, where sexual development is induced only in blood-fed females [5, 6]. Our previous study showed that coinfection with the gregarine *Ps. sergenti* does not have an apparent effect on the development of *Leishmania tropica* in *P. sergenti* [7].

It has been previously reported that the rearing conditions of larvae have an impact on the maintenance of parasites and their development in mosquito adults. It was shown that the quality of the larval diet changes the vector competence of *Anopheles stephensi* to *Plasmodium yoelii* [8], and that ambient temperature during the larval development of *Aedes albopictus* negatively correlates with the likelihood of adult infection by Chikungunya virus (CHIKV): females developed from larvae kept at 18 °C had higher infection rates than those from larvae kept at 24 and 32 °C [9]. As far as we are aware, similar studies have never been done in sand flies. Therefore, we studied the impact of rearing temperature of immature stages of the Old World sand fly *Phlebotomus sergenti* on the development of *Pychodiella sergenti* and the susceptibility of adult sand flies to *Leishmania tropica*.

## Methods

### Sand flies and parasites

Two groups of *P. sergenti* (from a colony originating from adults caught in Amnun, Israel) infected with the gregarine *Ps. sergenti* were used in the study: (i) immature stages (eggs, larvae, pupae) maintained at 27 °C; and (ii) immature stages (eggs, larvae, pupae) maintained at 32 °C. Adults of both groups and experimentally infected females were kept at 26 °C. Other parameters of sand fly maintenance were as described by Volf & Volfova [3]. *Leishmania tropica* SU23 (MHOM/TR/98/HM) was maintained at 23 °C on M199 medium (Sigma-Aldrich, St. Louis, MO, USA) supplemented with 20 % foetal calf serum (Gibco, Life Technologies, Carlsbad, CA, USA), 1 % BME vitamins (Sigma-Aldrich, St. Louis, MO, USA), 2 % filtered human urine and amikacin (250 μg/ml).

### Experimental infections with *Leishmania tropica*

Sand fly females were membrane-fed on suspension of heat-inactivated rabbit blood containing 1 × 10^6^ promastigotes/ml. Blood-fed females were maintained at 26 °C. On days 2 and 7–9 post-blood meal, females were dissected under a stereomicroscope and checked for the intensity and localization of infections using a compound light microscope. Intensities of infection were graded according to Myskova et al. [10] as weak (less than 100 promastigotes/gut), moderate (100–1,000 promastigotes/gut) and heavy (more than 1,000 promastigotes/gut). Data from two independent experiments were pooled and evaluated statistically by means of the Chi-square test using STATISTICA 12.0 (StatSoft Inc., Tulsa, OK, USA).

### Gregarine infection

Different sand fly stages, namely the actively feeding fourth-instar larvae (before defecation of the midgut content) and sugar-fed adults of both sexes (1, 4 and 7 days post-eclosure) were dissected under the stereomicroscope and checked for the presence and number of gregarines. Results were evaluated statistically by the Chi-square and Kruskal-Wallis tests using STATISTICA 12.0.

### Morphometric analysis

Measurements of wing length or wing veins are often used to determine the size of adult mosquitoes and sand flies [11, 12]. Therefore, wings of emerged females were dissected from the body and mounted on slides using CMCP-10 mounting medium (Polysciences, Inc., Warrington, PA, USA). Slides were observed under an Olympus BX51 microscope and photographed with Olympus D70 camera software. The effect of temperature was evaluated by measuring the length of the R2, R3, R4, R5, M1 and M2 wing veins, as previously described by Belen et al. [12]. ANOVA was used for statistical evaluation using STATISTICA 12.0.

## Results

### Morphometric analysis

**Fig. 1:**
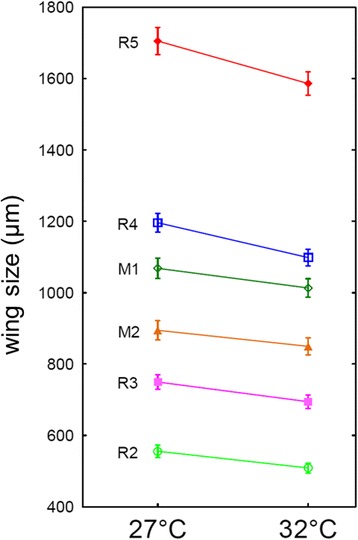
Wing size of *P. sergenti* females depending on temperature. The association between ambient temperature during immature stage development and the wing size of adult *P. sergenti* females. Females from larvae reared at 27 °C were significantly larger than those from 32 °C. *Abbreviations*: M1, Media 1; M2, Media 2; R2, Radius 2; R3, Radius 3; R4, Radius 4; R5, Radius 5

Larval rearing temperature significantly affected the size of adult sand flies. Measures of all wing veins studied were significantly longer in females originating from larvae developed at 27 °C than those from 32 °C (ANOVA: *F*_(6,55)_ = 13.26, *P* < 0.001). Data from morphometric analysis are shown in Fig. 1. As expected, temperature also affected the rate of development: the average time interval from egg-laying to emergence of adults was 36 days at 27 °C compared to 26 days at 32 °C.

### Effects of immature stage rearing temperatures on experimental infections with *Leishmania tropica*

**Fig. 2:**
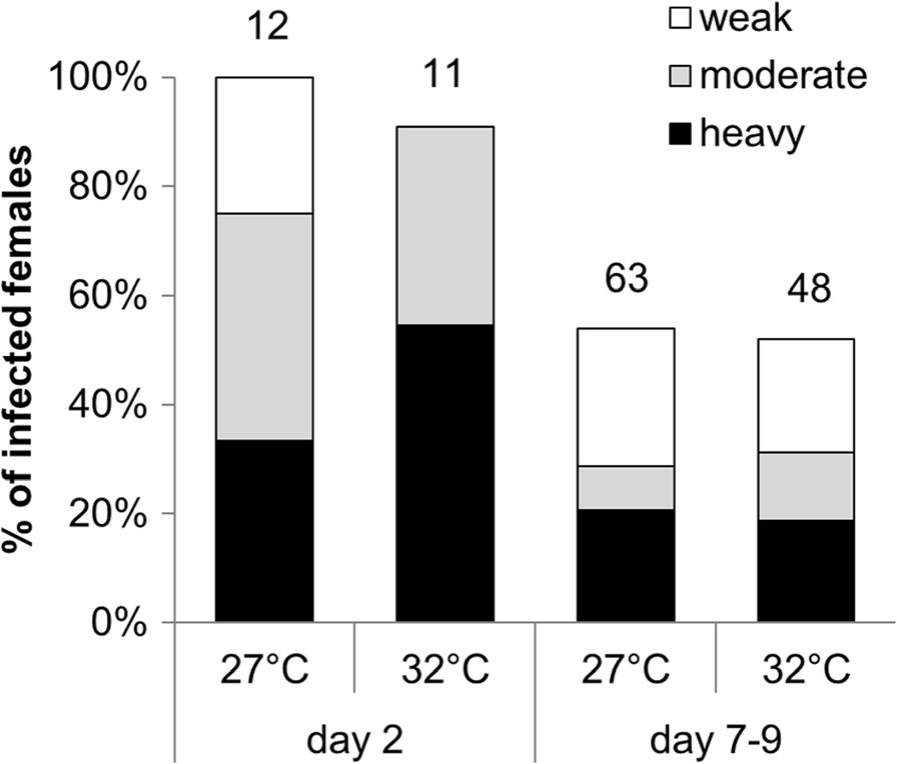
Development of *L. tropica* in *P. sergenti*. There were no significant differences in infection rates or infection intensity of *L. tropica* in *P. sergenti* females emerged from larvae kept at 27 °C or 32 °C. Numbers of dissected females are shown above the bars

Larval rearing temperature did not affect the infection rates or intensities of *L. tropica* infections in *P. sergenti* females (Fig. 2). No significant differences were found in infection rates (day 2: *χ* ^2^ = 1.14, *df* = 1, *P* = 0.29; days 7–9: *χ* ^2^ = 0.04, *df* = 1, *P* = 0.84) or in intensities of *Leishmania* infection (day 2: *χ* ^2^ = 3.36, *df* = 2, *P* = 0.19; days 7–9: *χ* ^2^ = 0.85, *df* = 2, *P* = 0.65).

### Effects of immature stage rearing temperatures on gregarine infections

The rearing temperature of immature stages of *P. sergenti* sand flies infected by *Ps. sergenti* had a marked effect on infection rates. All 117 control sand flies tested (60 males and 57 females derived from larvae kept at 27 °C) were infected with *Ps. sergenti* gamonts, with a mean number of 29.5 gamonts per individual. On the other hand, sand flies produced from larvae kept at 32 °C were almost completely gregarine-free: only three of 120 sand flies dissected (60 males and 60 females) were infected by gregarines, with a mean number of 0.04 gamonts per individual. The differences between groups were highly significant (*χ* ^2^ = 225.29, *df* = 1, *P* < 0.0001). To investigate in which developmental stage the gregarines disappeared, an additional 32 (kept at 27 °C) and 33 (kept at 32 °C) actively feeding (before defecation of the midgut content) fourth-instar larvae were dissected. Larvae kept at 27 °C were significantly more infected (*χ* ^2^ = 6.42, *df* = 1, *P* < 0.01) and the intensity of infection was significantly higher (Kruskal-Wallis test, *F*_(1,41)_ = 12.07, *P* < 0.001).

## Discussion

Here we show that the rearing temperature of immature stages did not affect parameters of *Leishmania* infection in adults. Similar studies have been done in mosquitoes, but with contradictory results. There was no observation of a consistent impact of larval rearing temperatures (19, 25 and 31 °C) on the infection, transmission or dissemination of West Nile virus in adults of *Culex tarsalis* [13]. In contrast, *Aedes albopictus* adults obtained from larvae kept at 18 °C had two or six times higher infection rates by Chikungunya virus than adults from larvae kept at 24 and 32 °C, respectively [9]. As expected, larvae kept at higher temperature developed faster and yielded smaller adults. We found that these size differences in *P. sergenti* adults had no effect on the infection rate and intensity of *L. tropica* infections. In mosquitoes, the relationship between vector size and their susceptibility to infection has been studied by various authors, with different outcomes. Larger *Anopheles gambiae* females were significantly more infected by *Plasmodium yoelii nigerinesis* than smaller ones, but no such effect was found in *Anopheles stephensi* [14]. Similarly, larger *Ae. aegypti* females were more likely to be infected by Dengue virus than smaller ones [11], but the opposite was found by Alto et al. [15]. In two other studies, no effect of mosquito size was described on infection parameters by three different viruses [16, 17]. These findings suggest that there are no general rules across all vectors-parasites/pathogens for a relationship between vector size and infection parameters, but rather results are species specific.

Rearing temperatures of immature stages had major impacts on the intensity of infection and infection rate of gregarines. A significant difference was visible by the fourth-instar larvae: those reared at 27 °C were infected more and had higher intensities of infection compared to those reared at 32 °C. The difference was more pronounced in adults: gregarine gamonts were found in all sand flies developed from larvae reared at 27 °C but in only three adults from larvae reared at 32 °C. This finding seems to confirm a previous hypothesis that the pupal stage is the most limiting part of the *P. sergenti* life-cycle for gregarine survival [18].

We demonstrated that increased temperature eliminated infection by the gregarine *Ps. sergenti* quite efficiently. In our hands, this method was far more effective than the washing of eggs described by Poinar & Thomas [19]. This washing method only reduced the numbers of gregarines *Psychodiella chagasi* and *Ps. sergenti* in colonies of *Lutzomyia longipalpis* and *P. sergenti*, respectively, but had to be repeated again for almost every sand fly generation [18].

We hypothesize that the elimination of gregarines in larvae and pupae reared at 32 °C might be caused by several factors. The increased metabolism of sand flies maintained at higher temperatures [20] leads to a faster reconstruction of larval tissue in pupae, which might be too quick for gregarines. Increased temperatures may also act negatively directly on gregarines, as suggested previously in other gregarine-insect pairs. For example, the effect of temperature was tested on the gametocysts and oocysts of two species of gregarines: *Blabericola migrator* and *B. cubensis*. In both species, no gametocysts completed development or produced oocysts at 10 or 40 °C. Oocyst viability in *B. migrator* was highest at 18 °C (57 %) and 22 °C (77 %), and was markedly decreased at 27 °C (24 %) and 35 °C (2 %), with similar results for *B. cubensis* [21]. The temperature of 32 °C, used in our experiments, might have reduced the number of viable infective oocysts, which would have resulted in lower intensities of larval infections. Furthermore, suboptimal conditions during the pupal stage would result in a further decrease of parasite numbers in adult sand flies.

Another possible explanation of gregarine elimination in *P. sergenti* maintained at 32 °C is the enhanced immune response of the insect. Various beetles maintained at higher temperature display higher phenoloxidase and antibacterial activities [22, 23]. However, the immune response is not straightforwardly correlated with temperature, as individual components of the immune system possess different thermal optima and there is a complex network of interactions between temperature, time and origin of immune challenge. For instance, in *Anopheles stephensi* a maximum expression of nitric oxide synthase was found at 30 °C, while a peak of melanization, phagocytosis and defensin expression was observed at 18 °C [24]. Clearly, further experiments are necessary for an explanation of the mechanism of gregarine elimination in sand flies.

## Conclusions

Understanding the biotic and abiotic factors affecting parasite-vector interactions is crucial for predicting the spread of vector-borne diseases and their epidemiology. To our knowledge, this is the first study in sand flies to evaluate the effect of larval rearing temperature on the consequent susceptibility of adults to *Leishmania*. In the natural parasite-vector combination *P. sergenti*/*L. tropica* we did not find any effect. However, increased temperature very efficiently eliminated infection by the gregarine *Ps. sergenti*, and appears to be a novel method for cleaning parasitic gregarines from sand fly colonies. Our results suggest that rearing immature stages at 32 °C is a simple and effective method to obtain gregarine-free colonies.

## References

[1] Killick-Kendrick R (1999). The biology and control of phlebotomine sand flies. Clin Dermatol.

[2] Maroli M, Feliciangeli MD, Bichaud L, Charrel RN, Gradoni L (2013). Phlebotomine sandflies and the spreading of leishmaniases and other diseases of public health concern. Med Vet Entomol.

[3] Volf P, Volfova V (2011). Establishment and maintenance of sand fly colonies. J Vector Ecol.

[4] Lantova L, Volf P. Mosquito and sand fly gregarines of the genus *Ascogregarina* and *Psychodiella* (Apicomplexa: Eugregarinorida, Aseptatorina) - Overview of their taxonomy, life cycle, host specificity and pathogenicity. Infect Genet Evol. 2014;28:616–27. 10.1016/j.meegid.2014.04.021 24797386

[5] Lantova L, Ghosh K, Svobodova M, Braig HR, Rowton E, Weina P (2010). The life cycle and host specificity of *Psychodiella sergenti* n. sp. and *Ps. tobbi* n. sp. (Protozoa: Apicomplexa) in sand flies *Phlebotomus sergenti* and *Ph. tobbi* (Diptera: Psychodidae). J Invertebr Pathol.

[6] Lantova L, Volf P (2012). The development of *Psychodiella sergenti* (Apicomplexa: Eugregarinorida) in *Phlebotomus sergenti* (Diptera: Psychodidae). Parasitology.

[7] Jancarova M, Hlavacova J, Volf P. The development of *Leishmania tropica* in sand flies (Diptera: Psychodidae): A comparison of colonies differing in geographical origin and a gregarine coinfection. J Med Entomol. 2015;52:1378–80. 10.1093/jme/tjv135 PMC4634311 26336272

[8] Moller-Jacobs LL, Murdock CC, Thomas MB (2014). Capacity of mosquitoes to transmit malaria depends on larval environment. Parasit Vectors.

[9] Westbrook CJ, Reiskind MH, Pesko KN, Greene KE, Lounibos LP (2010). Larval environmental temperature and the susceptibility of *Aedes albopictus* Skuse (Diptera: Culicidae) to Chikungunya virus. Vector Borne Zoonotic Dis.

[10] Myskova J, Votypka J, Volf P (2008). *Leishmania* in sand flies: comparison of quantitative polymerase chain reaction with other techniques to determine the intensity of infection. J Med Entomol.

[11] Sumanochitrapon W, Strickman D, Sithiprasasna R, Kittayapong P, Innis BL (1998). Effect of size and geographic origin of *Aedes aegypti* on oral infection with dengue-2 virus. Am J Trop Med Hyg.

[12] Belen A, Alten B, Aytekin AM (2004). Altitudinal variation in morphometric and molecular characteristics of *Phlebotomus papatasi* populations. Med Vet Entomol.

[13] Dodson BL, Kramer LD, Rasgon JL (2012). Effects of larval rearing temperature on immature development and West Nile virus vector competence of *Culex tarsalis*. Parasit Vectors.

[14] Takken W, Smallegange RC, Vigneau AJ, Johnston V, Brown M, Mordue-Luntz AJ (2013). Larval nutrition differentially affects adult fitness and *Plasmodium* development in the malaria vectors *Anopheles gambiae* and *Anopheles stephensi*. Parasit Vectors.

[15] Alto BW, Lounibos LP, Mores CN, Reiskind MH (2008). Larval competition alters susceptibility of adult *Aedes* mosquitoes to dengue infection. Proc R Soc London B.

[16] Kay BH, Edman JD, Fanning ID, Mottram P (1989). Larval diet and the vector competence of *Culex annulirostris* (Diptera: Culicidae) for Murray Valley encephalitis virus. J Med Entomol.

[17] Reisen WK, Hardy JL, Presser S (1997). Effects of water quality on the vector competence of *Culex tarsalis* (Diptera: Culicidae) for western equine encephalomyelitis (Togaviridae) and St. Louis encephalitis (Flaviviridae) Viruses. J Med Entomol.

[18] Lantova L, Svobodova M, Volf P (2011). Effects of *Psychodiella sergenti* (Apicomplexa, Eugregarinorida) on its natural host *Phlebotomus sergenti* (Diptera, Psychodidae). J Med Entomol.

[19] Poinar GO, Thomas GM, Thomas GM (1984). Bacteria. Laboratory guide to insect pathogens and parasites.

[20] Benkova I, Volf P (2007). Effect of temperature on metabolism of *Phlebotomus papatasi* (Diptera: Psychodidae). J Med Entomol.

[21] Kolman JA, Clopton ER, Clopton TD (2015). Effects of developmental temperature on gametocysts and oocysts of two species of gregarines *Blabericola migrator* and *Blabericola cubensis* (Apicomplexa: Eugregarinida: Blabericolidae) parasitizing blaberid cockroaches (Dictyoptera: Blaberidae). J Parasitol.

[22] Adamo SA, Lovett MM (2011). Some like it hot: the effects of climate change on reproduction, immune function and disease resistance in the cricket *Gryllus texensis*. J Exp Biol.

[23] Catalan TP, Wozniak A, Niemeyer HM, Kalergis AM, Bozinovic F (2012). Interplay between thermal and immune ecology: effect of environmental temperature on insect immune response and energetic costs after an immune challenge. J Insect Physiol.

[24] Murdock CC, Paaijmans KP, Bell AS, King JG, Hillyer JF, Read AF, et al. Complex effects of temperature on mosquito immune function. Proc R Soc London B. 2012;279:3357–66. 10.1098/rspb.2012.0638 PMC3385736 22593107

